# The Ecology, Evolution, Systematics, and Behaviour of Mites

**DOI:** 10.3390/ani14081142

**Published:** 2024-04-09

**Authors:** Maciej Skoracki, Monika Fajfer-Jakubek

**Affiliations:** 1 Department of Animal Morphology, Faculty of Biology, Adam Mickiewicz University, Uniwersytetu Poznańskiego 6, 61-614 Poznań, Poland; 2 Department of Molecular Biology and Genetics, Institute of Biological Sciences, Cardinal Stefan Wyszynski University, Wóycickiego 1/3, 01-938 Warsaw, Poland; m.fajfer@uksw.edu.pl

In the intricate web of biodiversity, mites serve as fundamental, yet often overlooked, architects, playing essential roles in ecosystems across the globe. Their interactions with plants, animals, and microorganisms highlight a complex array of ecological relationships that influence the distribution, diversity, and dynamics of biological communities. This Special Issue, entitled “The Ecology, Evolution, Systematics, and Behaviour of Mites”, assembles a collection of studies that advances our understanding of mites through detailed examinations of their coevolutionary relationships, taxonomic diversity, molecular biology, and ecological interactions. The contributions within this Special Issue not only shed light on the multifaceted nature of mites but also emphasize the importance of interdisciplinary approaches to unravelling the mysteries of these abundant arthropods.

In this Special Issue, the coevolutionary associations between mites and their hosts are explored through several studies [1,2,3,4,5], providing insights into the host–parasite relationship, phylogeny, and host specificity of mites. For instance, the discovery of a new scale-mite species from Robert’s Tree Iguana [1] not only enriches the taxonomic diversity of mites but also provides novel insights into the phylogenetic relationships within the Pterygosomatidae family. Similarly, the discovery of *Tinamiphilopsis temmincki* on the Tataupa Tinamou [2] contributes to the understanding of syringophilid mites’ evolutionary history and host–parasite dynamics, challenging assumptions about host specificity and evolutionary pathways. Research on *Demodex* in the mouflon [3] expands our comprehension of host–parasite relationships by shedding light on the evolutionary history and ecological interactions of these skin mites in wild populations. Lastly, the description of three new feather mite species from Brazilian parrots [4] and the study on parasitic mites of African barbets [5] reveal the specificity of mite–host relationships and contribute to the broader understanding of coevolutionary dynamics between mites and birds, highlighting the role of ecological and evolutionary processes in shaping host–parasite interactions.

Significant strides in the taxonomic revision of mite groups are presented in articles [6,7,8,9,10,11,12,13], showcasing the evolving nature of mite systematics. The taxonomy of the Teneriffiidae family is clarified [6], while new species groups within the *Tenuipalpus* sensu lato are proposed [7]. A remarkable new species of phthiracaroid mites from the Peruvian Andes [8] highlights the discovery of novel taxa in underexplored regions. The revision of the genus *Neoprotereunetes* [9,10] and a comprehensive review of the *Neoseiulus* species in China [11] have improved our understanding of these groups. Furthermore, the establishment of a new subfamily, Cunaxicaudinae [12], highlights the continuous discovery of novel morphological features and their implications for understanding mite evolution and systematics. Finally, the addition of the article on three new species of *Aceria* from China [13] further enriches the contributions to the field of mite taxonomy in this Special Issue.

The incorporation of molecular techniques into mite research [14,15,16,17,18,19] has revolutionized our understanding of mite evolution, genetic diversity, and phylogenetic relationships. Studies on the biogeography of *Fuscozetes fuscipes* [14], genetic diversity in quill mites [15], and the ontogeny and ecology of *Nanhermannia coronata* [16] have demonstrated the utility of DNA barcoding and molecular phylogenetics in uncovering hidden diversity and clarifying taxonomic relationships. Molecular identification of Laelapidae mites [17] and description of a new *Ultratenuipalpus* species [18] further illustrate the power of molecular data in advancing our understanding of mite biology. Nonetheless, at the same time, a survey of mite contamination in public genomic databases [19] reveals the widespread presence of mite DNA in sequencing projects, offering insights into mite diversity and host associations through unintended data sources.

Ecological studies [20,21,22] have explored the interactions between mites, their hosts, and the environment, revealing the adaptive strategies and ecological roles of mites in various habitats. While the former research on Uropodina mites in dormouse nest boxes [20] shed light on the niche preferences and community dynamics of mites in mammalian nests, the latter explores similar ecological dynamics in bird nests, illustrating how mites adapt to and exploit these specialized niches. Research on the life-type characteristics of three spider mite pests [21] complements these insights by demonstrating the influence of host plants on mite behaviour and life history strategies, emphasizing the adaptive nature of these pests to different environmental conditions. Collectively, from examining mite communities in nests [20,22] to assessing the impact of host plants on spider mite pests 21], these studies underscore the adaptive flexibility of mites and their intricate interactions with the surrounding world. Such research is essential for understanding the complex behaviours of mites and the ecological niches they inhabit.

All the articles in this Special Issue advance our knowledge across the spectrum of mite biology, from their ecological roles to their evolutionary dynamics. By integrating taxonomic revisions, molecular analyses, and ecological studies with investigations into host–parasite interactions, this body of research illuminates the complexity of mites’ life and their crucial roles within ecosystems. As we continue to explore the mysteries of the mite world, the interconnectedness becomes ever more apparent, highlighting the importance of interdisciplinary approaches in capturing the full scope of mite biodiversity and evolutionary interactions in the natural world.
